# Transcriptome analysis of differentially expressed genes and pathways associated with mitoxantrone treatment prostate cancer

**DOI:** 10.1111/jcmm.14100

**Published:** 2018-12-27

**Authors:** Sanqiang Li, Ruifang Li, Yu Ma, Cong Zhang, Tao Huang, Sha Zhu

**Affiliations:** ^1^ Key laboratory of Infection and Immunization, Department of Immunology, College of Basic Medical Sciences Zhengzhou University Zhengzhou Henan China; ^2^ Medical College Henan University of Science and Technology Luoyang Henan China; ^3^ College of Biological Engineering Henan University of Technology Zhengzhou Henan China; ^4^ Cancer Hospital Affiliated to Zhengzhou University Zhengzhou Henan China; ^5^ Collaborative Innovation Center of Cancer Chemoprevention Zhengzhou Henan China

**Keywords:** Castration‐resistant prostate cancer, differentially expressed genes, drug‐resistance, microarray, mitoxantrone

## Abstract

The global physiological function of specifically expressed genes of mitoxantrone (MTX)‐resistant prostate cancer (PCa) is unclear. In this study, gene expression pattern from microarray data was investigated for identifying differentially expressed genes (DEGs) in MTX‐resistant PCa xenografts. Human PCa cell lines DU145 and PC3 were cultured in vitro and xenografted into severe combined immunodeficiency (SCID) mice, treated with MTX intragastrically, three times a week until all mice relapsed. Gene expression profiles of the xenografts from castrated mice were performed with Affymetrix human whole genomic oligonucleotide microarray. The Cytoscape software was used to investigate the relationship between proteins and the signalling transduction network. A total of 355 overlapping genes were differentially expressed in MTX‐resistant DU145R and PC3R xenografts. Of these, 16 genes were selected to be validated by quantitative real‐time PCR (qRT‐PCR) in these xenografts, and further tested in a set of formalin‐fixed, paraffin‐embedded and optimal cutting temperature (OCT) clinical tumour samples. Functional and pathway enrichment analyses revealed that these DEGs were closely related to cellular activity, androgen synthesis, DNA damage and repair, also involved in the ERK/MAPK, PI3K/serine‐threonine protein kinase, also known as protein kinase B, PKB (AKT) and apoptosis signalling pathways. This exploratory analysis provides information about potential candidate genes and may bring new insights into the molecular cascade involvement in MTX‐resistant PCa.

## 1 INTRODUCTION

Prostate cancer (PCa) is the most common cancer in men in western countries.^1^ The majority of patients with advanced PCa have disease that is initially sensitive to androgen deprivation therapy (ADT), which successfully reduces tumour burden, improves symptoms and can delay disease progression for several years,^2^, ^3^ although, responses are generally not durable and disease progression is inevitable. Mitoxantrone (MTX), a synthetic anthracenedione, has been routinely used for the treatment PCa for its palliative benefit which enhances clinical remission of the PCa patients. However, despite their initial response and survival benefits, the majority of patients eventually develop resistance to these therapies.^4^

The antineoplastic activity of MTX is believed to be related to its ability to bind DNA and inhibit DNA topoisomerase II, an essential enzyme in DNA synthesis and meiotic division which is highly expressed in cancer cells.^5^ Damage to DNA is a notable inducer of both transient and permanent alterations in cellular phenotypes. The accumulation of DNA lesions leads to genomic instability through chromosomes breaks, amplification of oncogenes and inactivation of tumour suppression genes, driving to the acquisition of a malignant cancer phenotype.^6^ However, cancer cells can overcome DNA damage by induction of a DNA damage secretory program such as proliferation, invasion, metastasis, especially treatment resistance can develop through a variety of signal pathways, including base excision repair, nucleotide excision repair, mismatch repair, direct repair and recombinational repair.^7^, ^8^ Comparative genomic hybridization can help to identify relevant genes involved in tumour chemotherapy‐resistance and to predict response and cancer prognosis.

In this study, the diversity and magnitude of transcriptional responses to genotoxic damage induced by MTX were assessed in two castration‐resistant prostate cancer (CRPC) xenografts types and their controls using gene expression profiling. Identification of up‐ and down‐regulated gene expression levels in MTX‐resistant CRPC could facilitate improved screening and understanding the underlying mechanisms of MTX resistance, paving the way for the development of targeted interventions that can circumvent such resistance to treatment.

## 2 MATERIALS AND METHODS

### 2.1 Cell viability assay prepared for SCID mice inoculation

Prostate cancer cell lines DU145 and PC3 were provided kindly by Fred Hutchinson Cancer Research Center. The cells were seeded in 96‐well plates in quintuplicate with Dulbecco's Modified Eagle Medium (DMEM, Invitrogen, Carlsbad, USA) basal medium plus 10% foetal bovine serum (FBS) and 1% penicillin/streptomycin, at 37°C in a humidified atmosphere with 5% CO_2_. After 24 hours of culture, cells were treated with MTX (0.01, 0.1, 1 or 10 mg/mL) for 24, 48 and 72 hours respectively. Cell medium was removed and 100 mL/well of MTT solution (0.5 mg/mL in PBS) were added and incubated for 3 hours (at 37°C, protected from light). After the end of incubation, the supernatants were removed carefully, 150 mL of dimethyl sulfoxide was added to each well. The cells were then shook for 10 minutes in the dark. Absorbance was measured at 450 nm in a Microplate Reader (Bio‐Rad 680). Analysis of the obtained results was done using GraphPad Prism 4 computer program to evaluate cell proliferation rate and cytostatic rate. Untreated cells were used as controls. For in vivo experiment, DU145 and PC3 cells were cultured in DMEM supplemented with 10% charcoal‐stripped FBS (Hyclone) and 1% penicillin/streptomycin at 37°C in a humidified atmosphere with 5% CO_2_. Confluent cells were harvested with trypsin‐ethylenediaminetetraacetic acid (EDTA) (0.05% trypsin and 0.53 mmol/L tetrasodium EDTA), centrifuged 5 minutes at 225 *g* and resuspended in the medium at 1 × 10^7^/mL single cells. Aliquots of 0.1 mL were used for subcutaneous injection into CB‐17 severe combined immunodeficiency (SCID) mice (purchased from Guangzhou Provincial Medical Experimental Center).

### 2.2 Tumour inoculation and treatment

The animal study was carried out in a specific pathogen‐free room and was approved by the Medical Ethics Committee of the Zhengzhou University in accordance with the Guide for the Care and Use of Laboratory Animals (NIH publication no. 80‐23, revised 1996). Four to six weeks old CB‐17 male SCID mice were used in the experiment. Cells (1 × 10^6 ^cells) were injected subcutaneously into both flanks resulting in two tumours per mouse to test the MTX sensitivity. Once tumours became palpable, the mice were randomly divided into four treatment groups (six mice per group). In the first three groups, MTX was administered three times a week at 0.35 mg/kg, 1 mg/kg and 3.5 mg/kg respectively. The fourth group was treated with physiological saline (control) at the same time‐points. In another set of experiment, animals with palpable tumours were also assigned into four groups: MTX (3.5 mg/kg), castration, MTX (3.5 mg/kg) in combination with castration and control. Surgical castration was performed after tumours have developed. MTX and saline were administered intragastriclly in a 100 µL volume three times a week in all experiments. The diameter of subcutaneously growing tumours was measured with a calliper twice a week until the animals were killed after 6 weeks of treatment. Tumour weight was calculated by the formula: Tumour weight (mg) = (length×width^2^)/2.

### 2.3 RNA extraction, Labelling, hybridization and scanning of microarray

Total tumour RNA was extracted using Trizol reagent (Takara, Dalian, China) and concentrations were determined by a spectrophotometer (NanoDrop, Nyxor Biotech). All the processes were carried out according to the manufacturers’ instructions. Enrichment of total RNA from samples was carried out using the RNeasy Micro kit (Qiagen, Germantown, MD, USA), and samples’ quality and quantity were assessed on a spectrophotometer. Hybridization was performed in Affymetrix Human Genome U133Plus2.0 Chambers. Washes and scanning of the arrays were carried out according to manufacturer's instructions. Images were autogridded and the chemiluminescent signals were quantified, corrected for background and spot and spatially normalized. Differentially expressed genes (DEGs) were identified through filtering the dataset using *P*‐value <0.01 and a signal‐to‐noise ratio>2 for use in anova statistical analysis.

### 2.4 Data preprocessing

The analysis was carried out using r package oligo (version 1.38.0, http://bioconductor.org/packages/release/bioc/html/oligo.html) to process the Affymetrix data files data by performing background correction, data normalization and expression calculation. Probes were annotated by annotation files, and without corresponding gene symbols the probes were removed. The DEGs between control and MTX‐treatment samples were screened by non‐paired *t* test in limma package.^9^ Genes within the threshold value |logFC (fold‐change)| >1 and *P*‐value <0.05 were identified as DEGs.

### 2.5 Functional and pathway enrichment analyses

DAVID (Database for Annotation, Visualization and Integrated Discovery http://david.abcc.ncifcrf.gov/, version 6.8)^10^ was used for Gene Ontology (GO) enrichment analysis. The overlapping DEG in MTX‐treatment xenografts DU145R and PC3R were screened out for functional enrichment. Gene Ontology enrichment analysis was used to predict the enrichment degree and the potential functions of the DEGs in biological processes (BP), cellular components (CC) and molecular functions. In addition, Kyoto Encyclopedia of Genes and Genomes (KEGG) pathway enrichment analysis was used for systematic analysis of gene functions indicating a statistically significant difference.

### 2.6 Pathway enrichment and network construction

Two hundred genes with significant differences were intercepted from differential gene expression data and statistically analyzed by the GeneSpring GX software package. The target gene expression data were analysed by Cytoscape and the signal pathway is derived from tumour‐related candidate genes in microarray data. In order to find out the DEGs closely related with signal pathway, additional filtering (minimum 3‐fold change) was applied to extract the most significant of these genes which were further analysed using Cytoscape software. Those genes with known gene symbols and their corresponding expression values were uploaded into the software. Networks of these genes were algorithmically generated based on their connectivity.

### 2.7 Western blot analysis

Preparation of total cell lysate and the procedures for Western blot analyses were performed. Protein samples were separated on 10% polyacrylamide resolving gels with the buffer system and transferred onto nitrocellulose membranes for 2 hours at 250 mA. Protein binding sites on the nitrocellulose were blocked for 1 hour at 25°C in 5% (w/v) Marvel/PBS/3% (v/v) Tween‐20 (PBST), then incubated overnight at 4°C with PARP1, ILB1, CDH1 and PLAUR monoclonal antibodies (1:1000 dilution; Invitrogen, California, USA). The membranes were washed 3 × 10 minutes in tris buffered saline tween and probed with horseradish peroxidise‐conjugated secondary antibodies (Amersham Life Sciences, Buckinghamshire, UK) for 1 hour at 25°C. Following 3 × 10 minutes washes in PBST, bands were detected using enhanced chemiluminescence (ECL+reagents, Amersham). Densitometric quantification of band intensities was performed with Kodak one‐dimensional image analysis software.

### 2.8 Gene validation by qRT‐PCR

Quantitative real‐time PCR was performed with QPK‐201 SYBR Green master mix (Toyobo, Osaka, Japan) and the ABI 7300 system from Applied Biosystems. The primers used in the study were obtained from Invitrogen (Beijing, China). Thermocycling parameters included a RT step at 50°C for 20 minutes, followed by a DNA polymerase activation step at 95°C for 2 minutes and 50 PCR cycles (95°C for 20 seconds, 60°C for 30 seconds). All reactions were conducted in triplicate. The fold‐change in differential expression for each gene was calculated using the comparative C_T _method.

### 2.9 Gene expression in tumour samples from patients

Tumour samples were collected from patients with metastatic PCa. We selected patients who were confirmed diagnostic of adenocarcinoma, evidence of progression despite castrate levels of testosterone, being eligible for systemic chemotherapy based on MTX. We were able to collect seven tumour samples embedded in 2 mL of optimal cutting temperature (OCT) medium, stored at −80°C until processing, and six samples formalin‐fixed, paraffin‐embedded (FFPE). These patients have been received transurethral prostatic resection before the start of MTX treatment.

Tissues from FFPE and OCT embedded were sectioned at 15 and 25 μm thicknesses, respectively, before the RNA extraction. Total RNA from FFPE samples was obtained using the GenElute™ FFPE RNA Purification Kit (Sigma). The RNAs from OCT samples were extracted using TRIzol Reagent (Invitrogen) according to the manufacturer's instructions. Quality and quantity of the total RNAs were measured by NanoDrop‐2000 Spectrophotometer (Nanodrop Technologies).

### 2.10 Statistical analysis

Results are presented as the mean ± SE of the mean. To determine whether differences between groups were statistically significant, Wilcoxon rank‐sum test of variance was performed, and *P < *0.05 was considered to indicate a statistically significant difference. spss 12.0 software was used for statistical analyses.

## 3 RESULTS

### 3.1 MTX sensitivity testing

**Figure 1:**
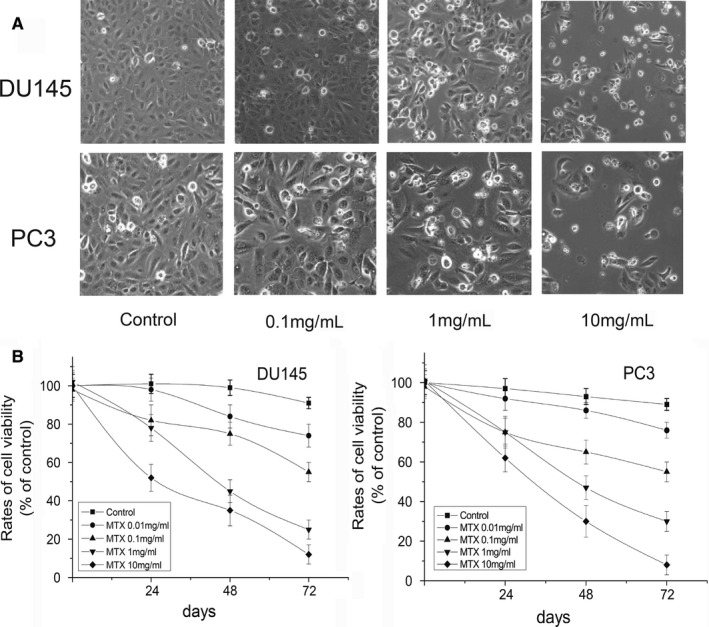
Cell morphology and viability of prostate cancer cells treated with different concentrations of mitoxantrone (MTX). A, DU145 cells: Controls (untreated cells), 0.1 mg/ml MTX, 1 mg/ml MTX, 10 mg/ml MTX; PC3 cells: Controls (untreated cells), 0.1 mg/ml MTX, 1 mg/ml MTX, 10 mg/ml MTX. B, Viability of DU145 and PC3 cells was determined by MTT assay. Error bars = SEM (n = 6)

**Figure 2:**
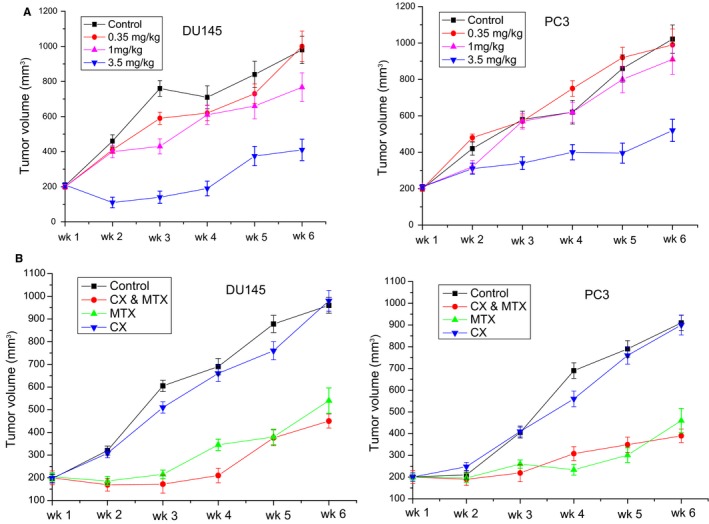
Effects of androgen deprivation and/or mitoxantrone (MTX) on prostate cancer (PCa) xenograft growth. A, Antitumour activities of different concentrations of MTX: Control, 0.35 mg/kg, 1 mg/kg and 3.5 mg/kg. B, Combined effects of androgen deprivation and MTX on PCa xenograft growth. (*P* < 0.015). (n = 5 mice per group)

DU145 and PC3 cells were cultured with increasing concentrations of MTX, at different time‐point, IC50 was determined by MTT assay. Results demonstrated that MTX decreased cell proliferation in a time and dose‐dependent manner. As shown in Figure 1A,B, the highest cytotoxicity of MTX was at 72 hours, and IC50 is 0.1 mg/mL for the PCa cells. To investigate MTX sensitivity in vivo, 10^6 ^cells of the PCa cells were inoculated into the flank region of 5‐week‐old male CB‐17 SCID mice to generate subcutaneous tumours. In general, palpable tumour formation started at 4‐5 weeks after subcutaneous implantation. Once tumours grew to nearly 200 mm^3^, mice were treated intragastrically with different concentrations of MTX. All animal procedures were performed according to local guidelines on animal care and with appropriate institutional certification. Tumour volume and weight were measured twice weekly and calculated by the formula as previously described. As shown in Figure 2A,B; tumour volumes differed significantly between control and MTX‐treated mice (*P < *0.01), while no significant effect was noted with less than 1 mg/kg of the drug administration. MTX decreased tumour growth in a dose‐dependent manner. Volume reduction for DU145 (45.8%) and PC3 (43.2%) xenografts was observed with 3.5 mg/kg MTX. However, all mice eventually relapsed and tumours became resistant to MTX.

To evaluate potential synergistic antitumour activities of androgen deprivation and MTX, SCID mice with palpable PCa xenografts were castrated and treated with 3.5 mg/kg MTX. We found that surgical castration did not significantly change the tumour size of either DU145 (*P = *0.731) or PC3 (*P = *0.794). However, as shown in Figure 2C,D; tumour volume was significantly decreased in the mice experienced with MTX (3.5 mg/kg) treatment (*P < *0.01). On day 28, the mean tumor volume of the xenografts in control group grew from 186.65 ± 32.84 to 712.72 ± 41.26 mm^3^, whereas experienced castration and MTX (3.5 mg/kg) treatment, the mean tumor volumes of DU145 and PC3 were 231.23 ± 24.52 and 254.47 ± 25.71 mm^3^ respectively (*P < *0.01). All mice eventually relapsed although consistently maintained their body weight during each study.

### 3.2 DEGs in MTX‐resistant xenografts

**Figure 3:**
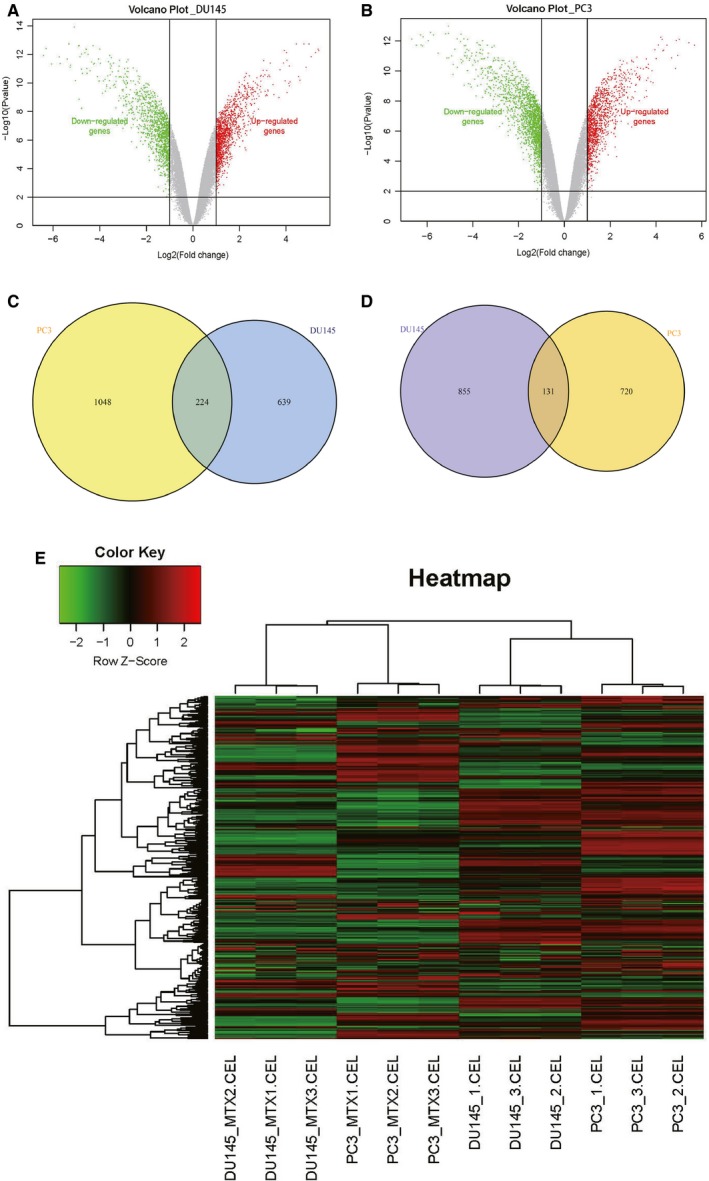
Volcano map and Venn diagram of differentially expressed genes (DEGs). A, Volcano plots of DEGs from DU145R. B, Volcano plots of DEGs from PC3R. Red spots represent up‐regulated genes, green spots represent down regulated genes, and grey dots represent non‐DEGs. C, VennPlot for the up‐regulated DEGs. D, VennPlot for the down‐regulated DEGs. E, Heatmap plot of the 355 overlapped genes between DU145R and PC3R dataset. Red represents higher expression and green represents lower expression. DEGs were selected by *P* < 0.05 and |log2 (fold‐change)| > 0.5. DEGs, differentially expressed genes

To identify the DEGs between MTX‐resistant xenografts and their controls, threshold |logFC| >1 and *P*‐value <0.05 were used in comparative analysis. A total of 1849, 2123 genes were extracted from the DU145R and PC3R respectively (Figure 3A,B). Among them, 986, 851 down‐regulated genes, and 863, 1272 up‐regulated genes were screened in the DU145R and PC3R xenografts respectively. We selected top 20 up‐regulated and down‐regulated genes according to the log ratio expression values (Tables 1 & 2), and among these, the ones in the MTX‐resistant groups whose expression levels were changed by more than 3‐fold compared with their control groups (*P* < 0.01). Upon comparison of the DEGs between both MTX‐resistant xenografts, 355 genes are overlapped, comprising 131 co‐downregulated genes and 224 co‐upregulated genes (Figure 3C,D). After that, the overlapping DEGs were clustered which can well differentiate the MTX‐treatment samples from the controls. The heatmap of the overlapping DEGs is shown in Figure 3E.

**Table 1:** Top 10 up‐and down‐expressed genes in the xenograft of DU145R vs its control

Primary accession	Gene symbol	Log2 ratio	Main function
NM_016192	TMEFF2	5.784	Function as both an oncogene and a tumour suppressor and may regulate prostate cancer cell invasion
NM_001618	PARP1	5.742	Involved in regulation of differentiation and proliferation and recovery of cell from DNA damage
NM_001005377	PLAUR	5.712	Acts as a receptor for urokinase plasminogen activator
NM_001565	CXCL10	5.532	Chemotactic for monocytes and T‐lymphocytes
NM_012253	TKTL1	5.208	Catalyses the transfer of a two‐carbon ketol group from a ketose donor to an aldose acceptor
NM_000361	THBD	4.793	Responsible for the conversion of protein C to the activated protein C
NM_007315	STAT1	4.769	Signal transducer and transcription activator
NM_001964	EGR1	4.545	Plays an important role in regulating the response to growth factors
NM_000442	PECAM1	4.217	Play a role in endothelial cell‐cell adhesion
NM_002585	PBX1	3.736	Acts as a transcriptional activator of PF4 in complex with MEIS1
NM_198381	ELF5	−3.163	Regulation of the later stages of terminal differentiation of keratinocytes and a number of epithelium‐specific genes
NM_020698	TMCC3	−3.703	May be involved in the regulation of the proteolytic processing of the amyloid precursor protein
NM_001001924	MTUS1	−4.546	Isoform 1 inhibits breast cancer cell proliferation, delays the progression and reduces tumour growth
NM_005242	F2RL1	−4.778	A member of the G‐protein coupled receptor 1 family of proteins
NM_001624	AIM1	−4.875	May function as suppressor of malignant melanoma.
NM_002276	KRT19	−5.091	Responsible for the structural integrity of epithelial cells
NM_023938	C1orf116	−5.257	Putative androgen‐specific receptor
NM_002354	EPCAM	−5.386	Functions as a homotypic calcium‐independent CAMs
NM_000165	GJA1	−5.695	A gap junction protein involved in synchronized contraction of the heart and in embryonic development
NM_144777	SCEL	−6.409	May function in the assembly or regulation of proteins in the cornified envelope

CAM, cell adhesion molecule.

**Table 2:** Top 10 up‐and down‐expressed genes in the xenograft of PC3R vs its control

Primary accession	Gene symbol	Log2 ratio	Main function
M_001005377	PLAUR	6.314	Acts as a receptor for urokinase plasminogen activator
NM_006888	CALM1	5.856	Mediates the control of a large number of enzymes through calcium‐binding
NM_003133	SRP9	5.484	Plays a critical role in role in targeting secretory proteins
NM_016192	TMEFF2	5.784	Function as both an oncogene and a tumour suppressor and may regulate prostate cancer cell invasion
NM_004613	TGM2	5.134	Catalyses the cross‐linking of proteins and the conjugation of polyamines to proteins
NM_004061	CDH12	4.678	Cadherins are calcium‐dependent cell adhesion proteins
NM_001114753	ENG	4.482	plays an important role in the regulation of angiogenesis
NM_001565	CXCL10	4.369	Chemotactic for monocytes and T‐lymphocytes
NM_003027	SH3GL3	3.987	Implicated in endocytosis
NM_001878	CRABP2	3.856	Transports retinoic acid to the nucleus
NM_005257	GATA6	−3.557	Involved in gene regulation specifically in the gastric epithelium
NM_005118	TNFSF15	−4.078	Mediates activation of NF‐kappa‐B
NM_005531	IFI16	−4.191	Involved in transcriptional regulation
NM_024915	GRHL2	−4.682	Transcription factor playing an important role in primary neurulation and in epithelial development
NM_004584	RAD9A	−4.923	inhibitor of zinc‐dependent metallocarboxypeptidases
NM_005797	MPZL2	−5.138	Mediates homophilic cell‐cell adhesion
NM_004004	GJB2	−5.347	Gap channels (gap junctions) are specialized cell‐cell contacts that provide direct intracellular communication
NM_144777	SCEL	−5.495	May function in the assembly or regulation of proteins in the cornified envelope
NM_001037330	TRIM16	−6.2	Play a role in the regulation of keratinocyte differentiation
NM_017697	ESRP1	−6.676	mRNA splicing factor that regulates the formation of epithelial cell‐specific isoforms

### 3.3 Functional and pathway enrichment analysis of overlapping DEGs

**Figure 4:**
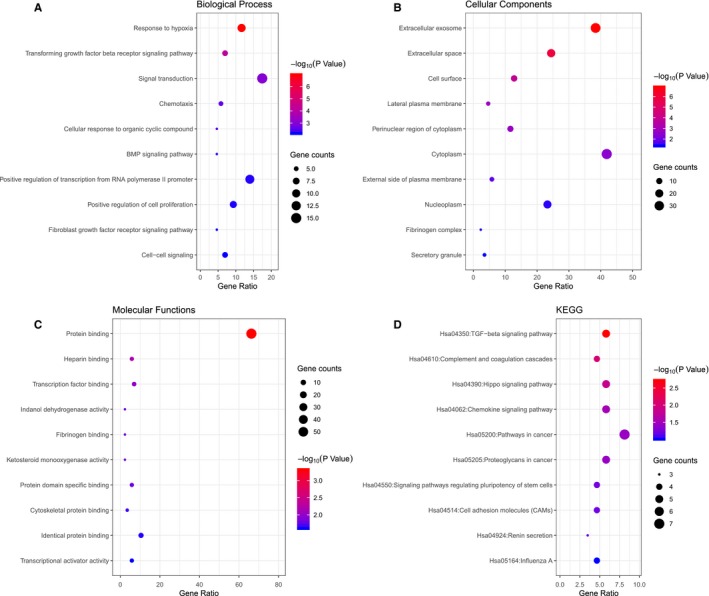
Gene Ontology‐enrichment analysis of overlapped differentially expressed genes. Here only show the top 10. A, Biological processes, B, Molecular functions, C, Cellular components, and D, Kyoto Encyclopedia of Genes and Genomes pathway analysis

Gene Ontology enrichment analysis revealed 355 overlapping genes that are involved in a number of BP including response to hypoxia, transforming growth factor β receptor signalling pathway, signal transduction and chemotaxis (Figure 4A). In terms of CC, DEGS were mostly enriched in the extracellular exosome, cell surface and lateral plasma membrane (Figure 4B). Molecular functions analysis indicated that the overlapping DEGs were mainly associated with protein binding, heparin binding and transcription factor binding (Figure 4C). Subsequential KEGG pathway enrichment analysis revealed that the common down‐regulated DEGs primarily enriched in the hippo signalling pathway, pathways in cancer, proteoglycans in cancer and cell adhesion molecules (CAMs) (Figure 4D). The top five enriched terms were presented in Table 3. These significantly enriched GO function and KEGG pathways could aid further understanding of the roles of these DEGs, involved in the development of MTX‐resistant CRPC.

**Table 3:** GO function and KEGG pathway enrichment analysis of DEGs

ID	Terms	Count	*P* value	Genes
Biological process				
GO:0001666	Response to hypoxia	10	1.12E‐07	KCNMA1, EGR1, CCL2, EPAS1, SMAD4, THBS1, ENG, SRF, PLAU, MB
GO:0007179	Transforming growth factor β receptor signalling pathway	6	7.24E‐05	CCL2, ID1, SMAD4, PARP1, GDF15, ENG
GO:0007165	Signal transduction	15	9.48E‐04	SH3GL3, EPAS1, CRABP2, TANK, PLAUR, CXCL10, THBD, CD274, IL1B, INPP4B, …
GO:0006935	Chemotaxis	4	2.62E‐03	ACKR3, PLAU, CXCL10, PLAUR
GO:0071407	Cellular response to organic cyclic compound	4	2.70E‐03	CCL2, IL1B, STAT1, ARHGDIA
Cellular components				
GO:0070062	Extracellular exosome	33	1.31E‐07	GALNT3, GNAI3, SLPI, PLAU, CDH1, CLDN11, EPCAM, TOR1A, IL1B, MB, …
GO:0005615	Extracellular space	21	1.23E‐06	ADNP, CXCL10, FBLN1, THBD, CST6, HIST2H2BE, TACSTD2, SERPINB5, SLPI, IL1B, …
GO:0009986	Cell surface	9	1.41E‐04	EPCAM, THBD, SLC1A3, ACKR3, AREG, THBS1, ALPP, FGFBP1, ENG
GO:0016328	Lateral plasma membrane	4	1.69E‐03	EPCAM, TACSTD2, CDH1, GJB2
GO:0048471	Perinuclear region of cytoplasm	9	1.72E‐03	GALNT3,VAMP8, CDH1, MAP7, NDRG1, ACKR3, LAMC2, PRKACB, STAT1
Molecular functions				
GO:0005515	Protein binding	57	4.87E‐04	FOSL2, ATP6AP2, TNNC1, CRABP2, SDC2, CXCL10, EPCAM, SPRED2, SERPINA1, PRKACB, …
GO:0008201	Heparin binding	5	6.73E‐03	CCL2, LAMC2, THBS1, FGFBP1, CXCL10
GO:0008134	Transcription factor binding	6	1.07E‐02	EPAS1, ID1, PBX1, ID3, PARP1, SRF
GO:0047718	Indanol dehydrogenase activity	2	1.40E‐02	AKR1C3, AKR1C1
GO:0070051	Fibrinogen binding	2	1.40E‐02	FBLN1, THBS1
KEGG pathyways				
hsa04350	TGF‐β signalling pathway	5	1.91E‐03	ID2, ID1, SMAD4, ID3, THBS1
hsa04610	Complement and coagulation cascades	4	9.42E‐03	THBD, SERPINA1, PLAU, PLAUR
hsa04390	Hippo signalling pathway	5	1.51E‐02	ID2, ID1, SMAD4, CDH1, AREG
hsa04062	Chemokine signalling pathway	5	2.99E‐02	GNAI3, CCL2, PRKACB, STAT1, CXCL10
hsa05200	Pathways in cancer	7	3.68E‐02	GNAI3, EPAS1, SMAD4, CDH1, LAMC2, PRKACB, STAT1

DEG, differentially expressed genes; GO, Gene Ontology enrichment analysis; KEGG, Kyoto Encyclopedia of Genes and Genomes.

### 3.4 Identification of candidate MTX‐resistant CRPC markers

**Figure 5:**
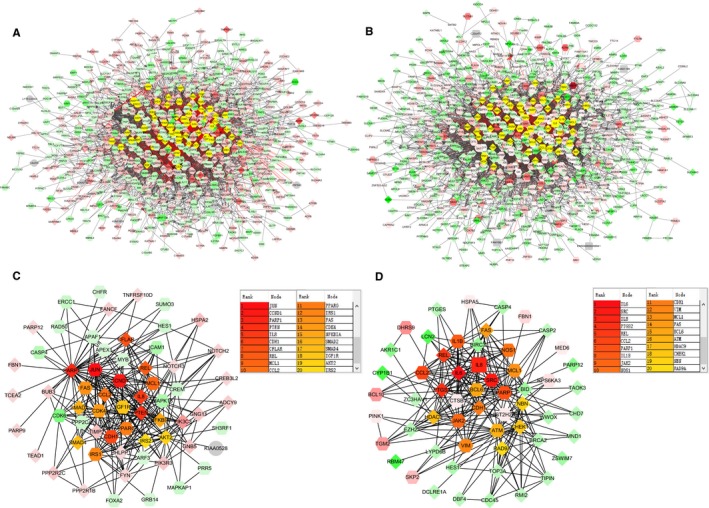
PPI networks constructed by the differentially expressed genes from mitoxantrone‐resistant xenografts. A, Network of significant proteins from DU145R. B, Network of significant proteins from PC3R. C, Network of hub significant proteins extracted from (A). D, Network of hub significant proteins extracted from (B). Red and green intensities indicate degree of up‐regulation and down‐regulation respectively. Genes in uncoloured notes were not identified as differentially expressed and were integrated into the networks indicating a relevance to this network

To further clarify the core genes of DEGs identified in the microarray analysis, protein‐protein interaction (PPI) networks were generated using DU145R and PC3R significant proteins. To characterize the properties of the hub nodes based on analysis of the PPI network, maximal clique centrality were chosen to identify candidate MTX‐resistant CRPC markers. A node was identified as a hub protein if its degree is more than 2‐fold of the median degree of all nodes and highlighted with yellow colour (Figure 5A,B). For the hub significant proteins, it consists of 63 nodes and 285 edges in the DU145R network, 58 nodes and 255 edges in PC3R network. By calculating the value of the three features for each hub significant protein, the median values of “Degree,” “Betweenness” and “Closeness” for DU145R and PC3R were 9.55, 784.2, 0.019 and 10.74, 808.5, 0.018 respectively. Functional annotation and pathway analysis of the nodes in the above networks are displayed in Table 4. After performing edge percolated component and shortest path analysis, the most significant modules composed of 10 nodes were screened out from the PPI networks and the hub genes in the networks with a connectivity degree >16 were identified (Figure 5C,D). By comparing the hub genes between both MTX‐resistant xenografts, PARP1, IL8 and CDH1 are overlapped.

**Table 4:** The pathways enriched for the MCC identified node in the PPI networks

ID	Terms	Count	*P* value	Genes
DU145R				
GO:0007219	Notch signalling pathway	4	3.68E‐03	HES1, NOTCH3, NOTCH2, CDK6
GO:0006351	Transcription, DNA‐templated	12	3.49E‐05	HES1, CCND1, MAPK13, CREM, JUN, PPARG, SMAD4, SMAD2, TCEA2, MYB, PARP1, APEX1
GO:0032000	Positive regulation of fatty acid β‐oxidation	3	2.57E‐04	IRS2, IRS1, AKT2
GO:0008286	Insulin receptor signalling pathway	4	5.33E‐04	IRS2, PIK3R3, IRS1, AKT2
GO:0046328	Regulation of JNK cascade	3	6.11E‐04	PHLPP1, IGF1R, SH3RF1
GO:2001275	Positive regulation of glucose import in response to insulin stimulus	3	1.11E‐03	PIK3R3, IRS1, AKT2
GO:0042127	Regulation of cell proliferation	5	3.26E‐03	FYN, TNFRSF10D, JUN, NFKBIA, FAS
GO:0045725	Positive regulation of glycogen biosynthetic process	3	1.11E‐03	IRS2, IRS1, AKT2
GO:0034097	Response to cytokine	3	4.50E‐03	REL, JUN, TIMP2
GO:0030513	Positive regulation of BMP signalling pathway	3	4.50E‐03	HES1, SMAD4, SMAD2
PC3R				
GO:0042127	Regulation of cell proliferation	9	4.71E‐08	BID, PTGS2, EZH2, BRCA2, BCL6, JAK2, CHEK1, FAS, SRC
GO:0071260	Cellular response to mechanical stimulus	4	2.92E‐04	BCL10, CHEK1, FAS, CASP2
GO:0071347	Cellular response to interleukin‐1	4	6.45E‐04	IL6, CCL2, PTGS2, PTGES
GO:0050767	Regulation of neurogenesis	3	8.27E‐04	NOS1, CHD7, BCL6
GO:0006954	Inflammatory response	6	1.04E‐03	CCL2, CASP4, PTGS2, REL, JAK2, FAS
GO:0000724	Double‐strand break repair via HR	4	1.14E‐03	NBN, ZSWIM7, BRCA2, ATM
GO:0045087	Innate immune response	6	1.33E‐03	BCL10, IL6, CASP4, REL, JAK2, SRC
GO:0097192	Extrinsic apoptotic signalling pathway in absence of ligand	3	3.94E‐03	MCL1, FAS, CASP2
GO:0070301	Cellular response to hydrogen peroxide	3	4.56E‐03	IL6, CYP1B1, EZH2
GO:0050727	Regulation of inflammatory response	3	8.72E‐03	CASP4, BCL6, JAK2

MCC, maximal clique centrality.

### 3.5 Validation of gene expression data by Western blotting and qRT‐PCR

**Figure 6:**
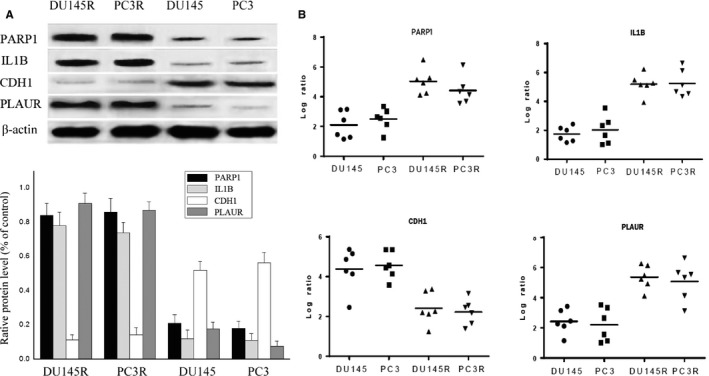
Expression profiles of PARP1, ILB1, CDH1 and PLAUR were evaluated by Western blot and qRT‐PCR. A, Western blot. B, Results expressed as western blotting band intensity. C, qRT‐PCR. Means ± SEM (n = 4)

**Figure 7:**
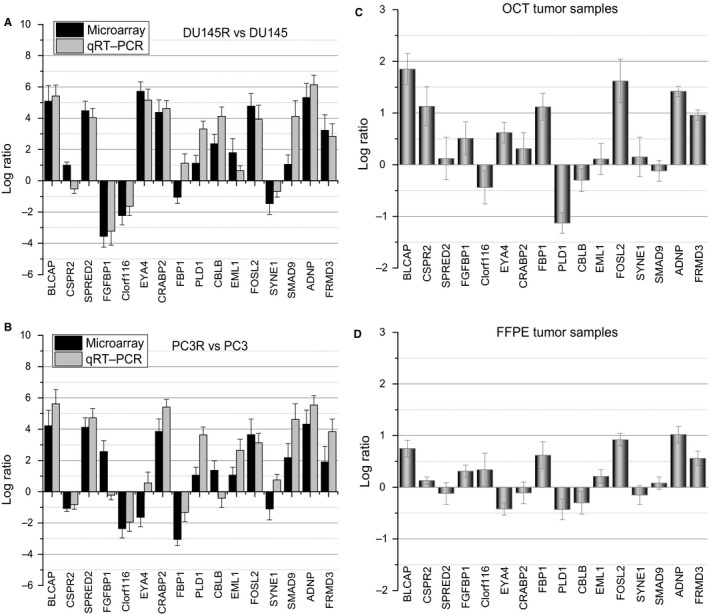
qRT‐PCR analysis of differentially expressed genes identified in the microarray. A, DU145R vs DU145, B, PC3R vs PC3, C, optimal cutting temperature (OCT) and D, formalin‐fixed, paraffin‐embedded (FFPE) tumour samples from patients with metastatic castration‐resistant prostate cancer. Expression data are represented by a log ratio calculated by comparing ΔCq from the xenograft with ΔCq from the controls. ΔCq was calculated as the difference between Cq of the targeted genes and Cq of the endogenous control gene ACTB

The expression patterns of four DEGs, PARP1, IL1B, CDH1 and PLAUR were evaluated by Western blot (Figure 6A) and quantitative real‐time PCR (qRT‐PCR) (Figure 6B). Results showed that up‐regulated ILB1 expression at the mRNA level, and enhanced positive expression of PARP1 and PLAUR in both DU145R and PC3R MTX‐resistant PCa xenografts. However, CDH1 was down‐expressed markedly in both MTX‐resistant tumour types as compared to their respective controls. In addition, a panel of 16 DEGs with the highest and lowest expression range in both androgen‐independent MTX‐resistant xenografts DU145R and PC3R vs their controls was selected and tested by qRT‐PCR (Figure 7A,B). Results showed that most of these genes exhibited a similar transcriptional profile to that of microarray data. The Pearson correction coefficient between the qRT‐PCR and microarray data for the 16 DEGs was 0.81. Hence, the microarray provided a reliable comparison of gene expression between androgen‐independent MTX‐resistant xenografts and untreated PCa.

Moreover, the 16 DEGs were also analysed in MTX‐resistant (n = 4) vs MTX‐sensitive (n = 3) OCT tumour samples, and MTX‐resistant (n = 5) vs MTX‐sensitive (n = 4) FFPE tumour samples from patients with metastatic CRPC (Figure 7C,D). This analysis showed that BLCAP and FOSL2 are significantly up‐regulated in OCT samples. Of the 16 DEGs studied, 12 genes in the OCT tumours and 10 genes in the FFPE tumours were deregulated in the same way as the xenograft models. Interestingly, BLCAP, FOSL2, ADNP and FRMD3 were concurrently up‐expressed in the androgen‐independent, MTX‐resistant xenografts, as well as in both FFPE and OCT samples from MTX‐resistant CRPC patients.

## 4 DISCUSSION

This study revealed global pathways and networks of DEGs involved in DU145R and PC3R MTX‐resistant PCa xenografts. Prostate cancer is a heterogeneous disease and many molecular methods have been used in the search to determine the mechanism behind its development, and find new therapeutic and prognostic targets.^11^ Microarray technology for gene expression profiling has proven to be successful in a variety of experimental settings having the potential to discover the diversified and dynamic molecules during tumour progression.^12^, ^13^ Despite enormous efforts made for differential expression detection and biomarker discovery, few methods have been investigated the gene expression level in tumour stage during MTX‐resistant progression. For this, our study attempt to identify putative molecules which may act as specific targets in cancer prognosis and therapy in future.

Our microarray study identified several hub genes and established their association with various genetic networks and biological pathways likely affecting MTX resistance in CRPC. Microarray analysed results revealed that PARP1, also known as a DNA nick sensor, overexpressed in DU145R and PC3R xenografts. Poly (ADP‐ribose) polymerase (PARP) is a family of 17 proteins involved in regulation of various cellular machineries, including necrosis, DNA repair, genomic stability, post‐translational modification of proteins and parthanatos.^14^, ^15^, ^16^ Recent analyses demonstrated that four of 17 human PARP (PARP1, PARP2, PARP4, PARP5) exhibit PARylating activity in vitro.^17^, ^18^ PARP activity is mainly due to PARP1 enzyme which detects DNA double‐strand breaks (DSBs) or DNA single‐strand breaks playing a role in protecting stalled replication forks from nuclease‐mediated degradation.^19^, ^20^ It was reported that PARP1 is also activated by some abnormal DNA structures or external signals through ERK pathway.^21^ Besides its canonical role in DNA repairing systems, PARP has demonstrated broader functions in controlling cell survival and death in modulating key components of angiogenesis in cancer cells.^22^ Inhibition of PARP1 is being exploited for the cancer treatment.^23^, ^24^ In fact, PARP inhibitors have showed an extremely promising anticancer treatment and are currently tested in phase I and II clinical trials in different solid tumours.^25^

Network analysis helps us obtain global and integrated molecular information about interactions among the significant DEGs. One important network was identified around the AKT2 genes. AKT2 is a putative oncogene encoding a protein belonging to the serine‐threonine protein kinase, also known as protein kinase B, PKB (AKT) subfamily of serine/threonine‐protein kinases, as well as a key node on the phosphatidylinositol 3‐kinase (PI3K/AKT) pathway which is recognized as a key pathway in carcinogenesis occurring commonly in diverse human cancer cells.^26^, ^27^ Previous studies have revealed that aberrant activation of PI3K/AKT pathway is also closely associated with the process of cancer metastasis.^28^ PI3K phosphorylates AKT and consequently facilitates tumourigenesis and cancer progression through its downstream targets.^29^ Report indicated that expressing active AKT can avoid apoptosis and checkpoint‐dependent cell cycle arrest due to suppression of homologous recombination (HR) and reliance on error‐prone NHEJ.^30^ Studies demonstrated that DNA damage activated AKT2 expression and subsequently conferred apoptotic resistance in ovarian cancer cells.^31^ At the same network, significantly decreased expression of CASP4, CASP2 and TNF‐receptor superfamily protein fas cell surface death receptor (FAS) was observed among the DEGs of MTX‐resistant xenografts.

In our study, Ataxia‐telangiectasia mutated kinase (ATM) was found to be highly up‐regulated in MTX‐resistant xenografts, and observed as a hub gene in the network. This gene is also highly expressed in non‐small cell lung cancer (NSCL) exposed to carbon ion irradiation.^32^ Ataxia‐telangiectasia mutated kinase, a member of PI3K/AKT family protein, its main function is to control the cell cycle progression following DNA damage, particularly DSBs.^33^ Reports indicated that MTX produce DNA cross‐links and DNA replication defects in tumour cells, once DNA damage occurs, ATM pathway for HR repair is activated.^34^ DNA repair is an essential prerequisite for the maintenance of genomic integrity and cellular viability.^35^ It is demonstrated that DNA‐damaging drugs (including MTX) can trigger an ATM‐dependent DNA damage response, leading to increased cytokine secretion and resistance to chemotherapy‐induced apoptosis.^36^ Report also revealed that ATM together with phosphotyrosine binding domain and a leucine zipper motif (APPL), a regulator of ATM phosphorylation, modulates DNA damage repair and consequently raises the survival of pancreatic carcinoma cells.^37^ In addition, ATM has been implicated in the control of RNA splicing that may play a role in genomic stability.^38^, ^39^

DNA‐damage response activates a secretory program that comprises a diverse spectrum of proteases, growth factors and, cytokines that have been shown to contribute to wound healing and altered immune responses.^40^, ^41^ Interleukin‐6 (IL‐6), at high concentrations, is found to protect cancer cells from therapy‐induced DNA damage, oxidative stress and apoptosis by facilitating the repair and induction of antioxidant response.^42^ In our study, IL6 is up‐regulated and screened as a hub gene in androgen‐independent, MTX‐resistant xenografts. Evidence support DNA damage and stress induce sustained IL‐6 and IL1B production in PCa through P2Y11 receptor‐p38 MAPK‐NF‐κB signalling pathway,^6^, ^43^ which may play an essential role in promoting cancer cells proliferation, survival, invasiveness and metastasis. Therefore, inhibition of IL‐6 or in combination with conventional anticancer therapies may be a potential therapeutic strategy for the treatment of MTX‐resistant PCa.

Sixteen DEGs were selected to be validated by qRT‐PCR according to their function and relative expression level in MTX‐resistant xenografts. These DEGs were further tested in MTX‐resistant CRPC patient samples (seven OCT and six FFPE samples). Results confirmed that BLCAP, EML1, FOSL2, ADNP and FRMD3 were deregulated in the same way among the xenografts, FFPE and OCT tumour samples. However, discordant results were observed in the expression of other genes such as PLD1, which was down‐expressed in MTX‐resistant tumour samples but was significantly overexpressed in MTX‐resistant xenografts. These conflicting results may be due to cellular heterogeneity between the xenografts from cell lines and the tumour sample from patients. Therefore, further clinical validation of these results is needed in a large cohort of patients.

In analysing, often individual genes were found in multiple categories of functions related to cancer development including cell signalling, cell death, cellular growth and proliferation. Besides, it reminds us there are certain limits in the analysis as there are many different gene interactions resulting from various cellular/experimental conditions. Nevertheless, this exploratory analysis may be still useful to bestow a theranostic perspective to the current trend of research in PCa or to develop targeted therapies to overcome MTX chemotherapy resistance.

## DATA AVAILABILITY

The data of the study have been deposited into the Research Data Deposit (http://www.researchdata.org.cn), with the Approval Number as RDDB2018000423.

## CONFLICTS OF INTEREST

The authors have no conflicts of interest to declare.

## AUTHORS CONTRIBUTIONS

All authors took part in writing, reviewing and editing the manuscript. S. Zhu, SQ Li and T. Huang designed experiments; RF Li, Y. Ma, C. Zhang, H. Wang, CJ Guo performed experiments; S. Zhu, Y. Ma, C. Zhang, T. Huang prepared figures; All authors reviewed the manuscript and approved it for publication.
